# Strabismus and Self-Perception: A Study on Self-Concept in Pediatric Strabismus Patients and the Impact of Surgical Intervention

**DOI:** 10.3390/children13081018

**Published:** 2026-07-31

**Authors:** Ceren Gürez, Didem Dizdar Yiğit, Asli İnal

**Affiliations:** 1 Department of Ophthalmology, Beyoglu Eye Research and Training Hospital, Istanbul 34421, Turkey; 2 Department of Ophthalmology, Marmara University Medicine Faculty, Istanbul 34854, Turkey

**Keywords:** self-concept, self-esteem, strabismus surgery

## Abstract

**Highlights:**

**What are the main findings?**

- Most school-aged children reported that strabismus negatively affected their daily lives and peer relationships.
- Self-perception of physical appearance improved six months after strabismus surgery, whereas overall self-concept remained largely unchanged.

**What are the implications of the main findings?**

- Assessment of psychosocial well-being should complement the clinical evaluation of children undergoing strabismus surgery.
- Patient-reported outcome measures, such as the Piers–Harris Self-Concept Scale, may provide valuable information beyond traditional ophthalmic outcomes.

**Abstract:**

**Introduction**: This study aimed to investigate the impact of strabismus on self-concept in school-aged children and to evaluate changes in self-concept six months after strabismus surgery. **Subjects and Methods**: This prospective multicenter study included children aged 7–15 years who underwent strabismus surgery at the Strabismus Units of Beyoğlu Eye Research and Training Hospital and Marmara University Hospital between December 2023 and June 2024. Self-concept was assessed using the validated Turkish version of the Piers–Harris Self-Concept Scale (PHSCS) before surgery and six months postoperatively. Only patients with complete preoperative and postoperative assessments were included in the final paired analysis. **Results**: A total of 116 children were enrolled, of whom 84 (42 boys and 42 girls; mean age, 10.9 ± 2.2 years) had complete data and were included in the final analysis. Overall, 73.6% of participants reported that strabismus affected their daily lives, with 77.4% describing this impact as negative. Furthermore, 65.5% stated that strabismus affected their relationships with friends. Following surgery, 80.9% of participants were satisfied with the surgical outcome, and 85.7% would recommend the procedure to others. The mean total PHSCS score increased from 58.2 ± 10.4 preoperatively to 59.0 ± 10.1 postoperatively, although this difference did not reach statistical significance (*p* = 0.05). In contrast, the Physical Appearance and Attributes subscale showed a significant improvement after surgery (8.3 ± 1.6 vs. 8.9 ± 1.9, *p* < 0.005), whereas no significant changes were observed in the remaining subscales. **Conclusions**: School-aged children with strabismus may experience psychosocial challenges related to their condition, particularly in their interactions with peers. Although overall self-concept remained largely unchanged, self-perception of physical appearance improved following strabismus surgery. These findings suggest that surgical correction may contribute to improvements in selected aspects of self-concept; however, larger prospective controlled studies are needed to confirm these observations.

## 1. Introduction

School age is a critical period in child development during which self-concept, social competence, emotional regulation, and interpersonal relationships are actively shaped. According to UNICEF, mental health problems and psychosocial difficulties affect a substantial proportion of children worldwide and may negatively influence academic achievement, peer relationships, and long-term well-being if not recognized and addressed early [1]. As many psychological and behavioral patterns established during childhood persist into adolescence and adulthood, early identification of modifiable risk factors that may adversely affect children’s psychosocial development is strongly recommended [2,3].

Strabismus is one of the most common ocular disorders in childhood and is characterized by ocular misalignment that may impair binocular vision and stereopsis. Beyond its functional and visual consequences, strabismus represents a visible facial difference that may influence how children perceive themselves and interact with others. Since the eyes play a central role in non-verbal communication, ocular misalignment may interfere with eye contact, facial expression, and social interactions, potentially leading to peer rejection, teasing, reduced self-confidence, and emotional distress. Previous studies have demonstrated that children with strabismus experience poorer health-related quality of life, greater social anxiety, and lower self-esteem than their visually healthy peers. Furthermore, the psychosocial burden of strabismus extends beyond affected children and has also been shown to influence parental anxiety and family quality of life.

Several studies have reported improvements in quality of life, psychosocial functioning, and parental satisfaction following strabismus surgery [1,4]. However, most previous investigations have primarily focused on health-related quality of life or parent-reported outcome measures rather than children’s own perceptions of themselves [5,6,7]. Although quality-of-life questionnaires provide valuable information regarding the impact of disease on daily functioning, they do not directly assess children’s internal perception of their own abilities, appearance, and social acceptance. Consequently, important aspects of children’s psychological development may remain underrecognized when self-perception is not evaluated directly.

Self-concept refers to an individual’s perception and evaluation of himself or herself across multiple domains, including physical appearance, social acceptance, academic competence, emotional well-being, and overall self-worth. Unlike general quality-of-life instruments, self-concept scales specifically measure how children perceive themselves during a developmental period in which identity and self-esteem are still evolving. The Piers–Harris Self-Concept Scale (PHSCS) is one of the most widely used and validated instruments for evaluating self-concept in school-aged children, providing both an overall self-concept score and domain-specific assessments of behavior, school status, physical appearance, anxiety, popularity, and happiness [8,9,10]. Despite its widespread use in pediatric psychology, only a limited number of studies have applied the PHSCS to children with ophthalmological disorders, and evidence regarding changes in self-concept following strabismus surgery remains scarce.

To our knowledge, no previous prospective multicenter study has evaluated changes in self-concept using the validated Turkish version of the Piers–Harris Self-Concept Scale before and after strabismus surgery in school-aged children. Therefore, the present study aimed to investigate the impact of strabismus on children’s self-concept and to evaluate changes in self-perception six months after surgical correction. We hypothesized that successful surgical treatment would be associated with improvements in specific domains of self-concept, particularly those related to physical appearance and social functioning.

## 2. Subjects and Methods

This multicenter prospective study was conducted in accordance with the principles of the Declaration of Helsinki and was approved by the local ethics committee (Approval No. I09.2023.1776). Children aged 7 to 15 years who underwent strabismus surgery at the strabismus units of Beyoğlu Eye Research and Training Hospital and Marmara University Hospital between December 2023 and June 2024 were eligible for inclusion. Written informed consent was obtained from the parents or legal guardians of all participants. In addition, written assent was obtained from children aged 7 years and older before enrollment.

Comprehensive ophthalmological examinations, including cycloplegic refraction, best-corrected visual acuity, slit-lamp biomicroscopy, dilated fundus examination, ocular motility assessment, and angle of deviation measurements, were performed preoperatively and at the 6-month postoperative visit. Angle of deviation was measured using the alternate prism cover test at both distance (6 m) and near (33 cm).

The inclusion criteria were: (1) undergoing strabismus surgery during the study period; (2) ability and willingness to complete the study questionnaires; and (3) attendance at the scheduled postoperative follow-up. Exclusion criteria included incomplete ophthalmological records, missing questionnaire data, ocular diseases other than refractive error and strabismus, previous ocular surgery or trauma, and systemic neurological disorders. Of the 116 eligible children initially enrolled, 84 had complete preoperative and postoperative assessments together with complete ophthalmological records and were therefore included in the final paired statistical analyses. Participants with incomplete clinical records and/or missing questionnaire data were excluded from the final analysis. All surgical procedures were performed by experienced strabismus surgeons using standard limbal surgical techniques.

For the assessment of patients’ self-concept, four Likert-type questions were asked: “Does being strabismic affect your life?” “Does being strabismic affect your relationship with friends?” “Are you happy with the result of your surgery?”, and “Would you recommend surgery to a friend?”

Self-concept was evaluated using the validated Turkish version of the Piers–Harris Children’s Self-Concept Scale (PHSCS), a 60-item self-report questionnaire with dichotomous (“Yes”/”No”) responses. The scale provides both a total self-concept score and six domain-specific subscale scores assessing Behaviour, Intellectual and School Status, Physical Appearance and Attributes, Anxiety, Popularity, and Happiness and Satisfaction.

### Statistical Analysis

Descriptive statistics of the data were presented as mean, standard deviation, median, minimum, maximum, frequency, and percentage values. The distribution of the variables was assessed using the Kolmogorov–Smirnov and Shapiro–Wilk tests. Independent sample *t*-test and Mann–Whitney U test were used to analyze quantitative independent data. The Wilcoxon test was used in the analysis of dependent quantitative data. The chi-square test was used to analyze qualitative independent data, and the Fisher test was used when the chi-square test conditions were not met. Statistical analyses were performed using the SPSS 28.0 software (SPSS Inc., Chicago, IL, USA).

## 3. Results

**Figure 1:**
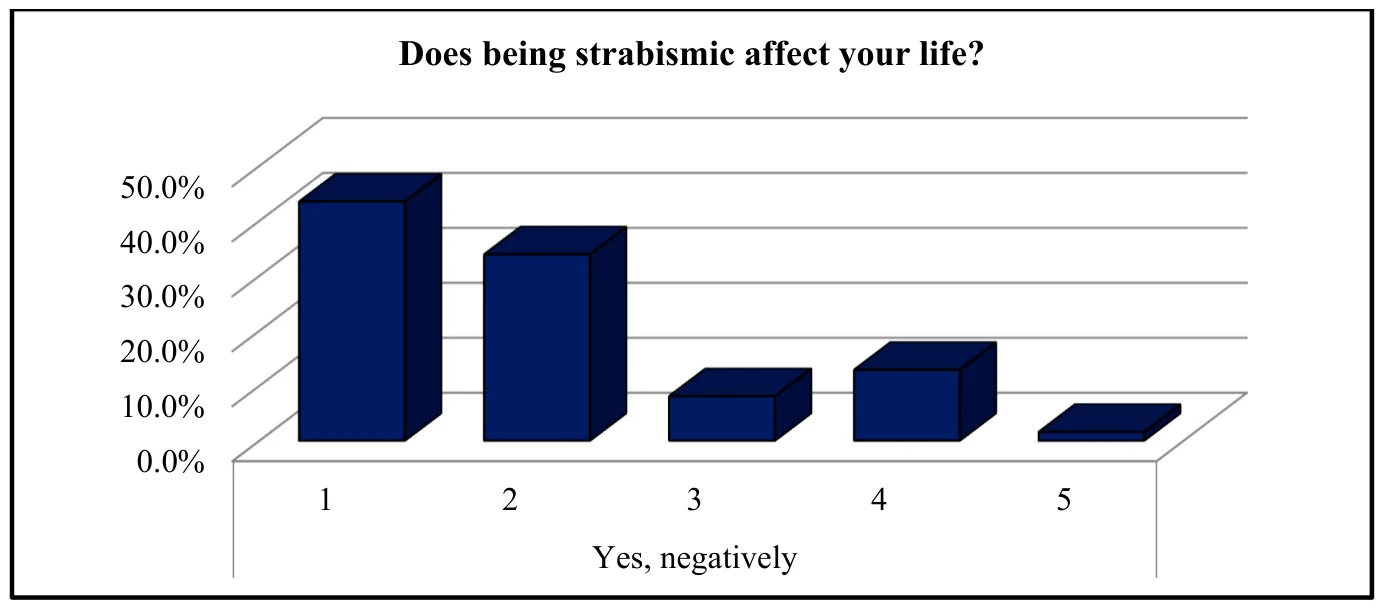
Evaluation of the impact of strabismus on the child’s life. 1: Totally agree; 2: Agree; 3: Not sure; 4: Disagree; 5: Totally disagree.

**Figure 2:**
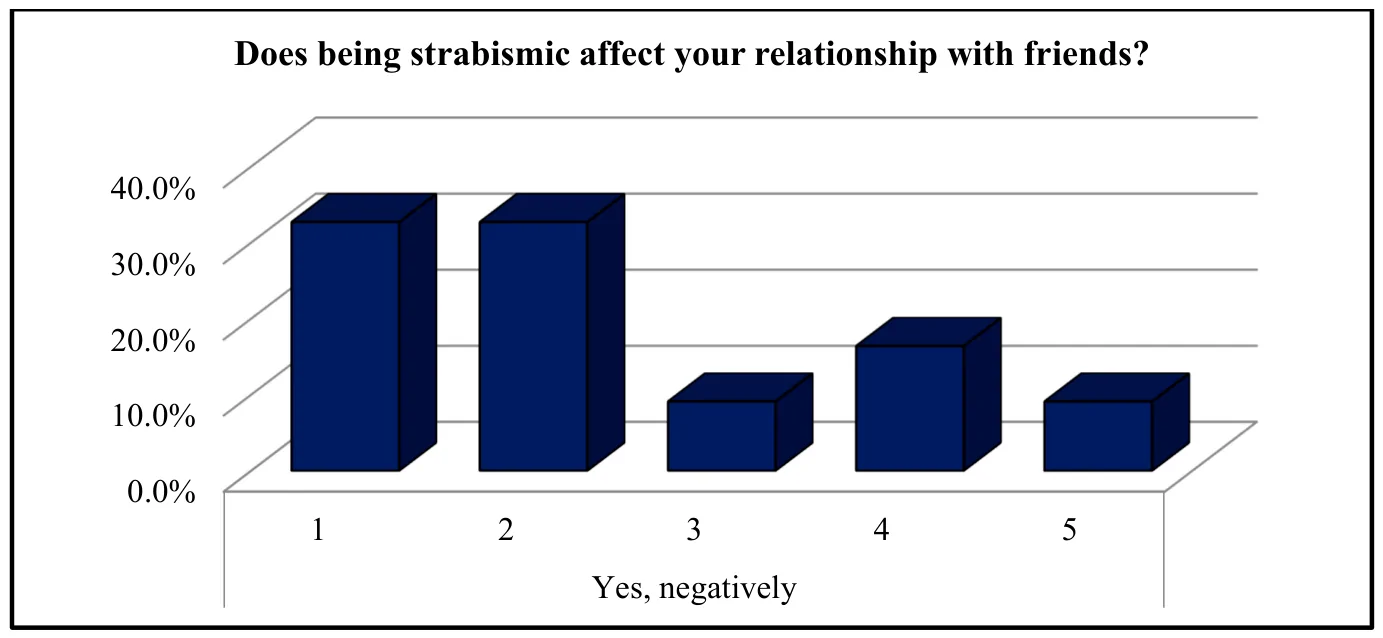
Evaluation of the effect of strabismus on the child’s relationships with friends. 1: Totally agree; 2: Agree; 3: Not sure; 4: Disagree; 5: Totally disagree.

**Figure 3:**
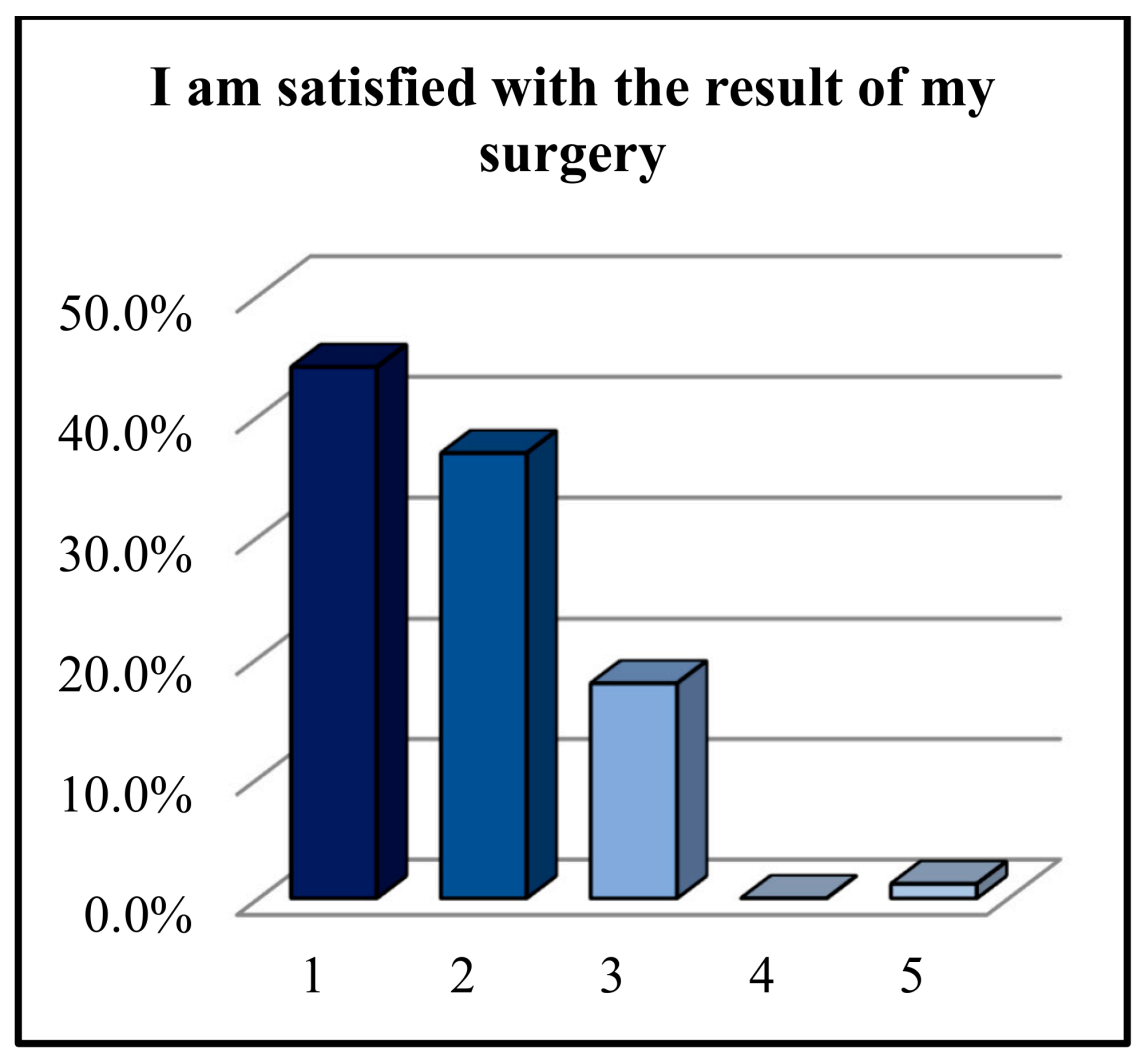
Evaluation of surgical outcome. 1: Totally agree; 2: Agree; 3: Not sure; 4: Disagree; 5: Totally disagree.

**Figure 4:**
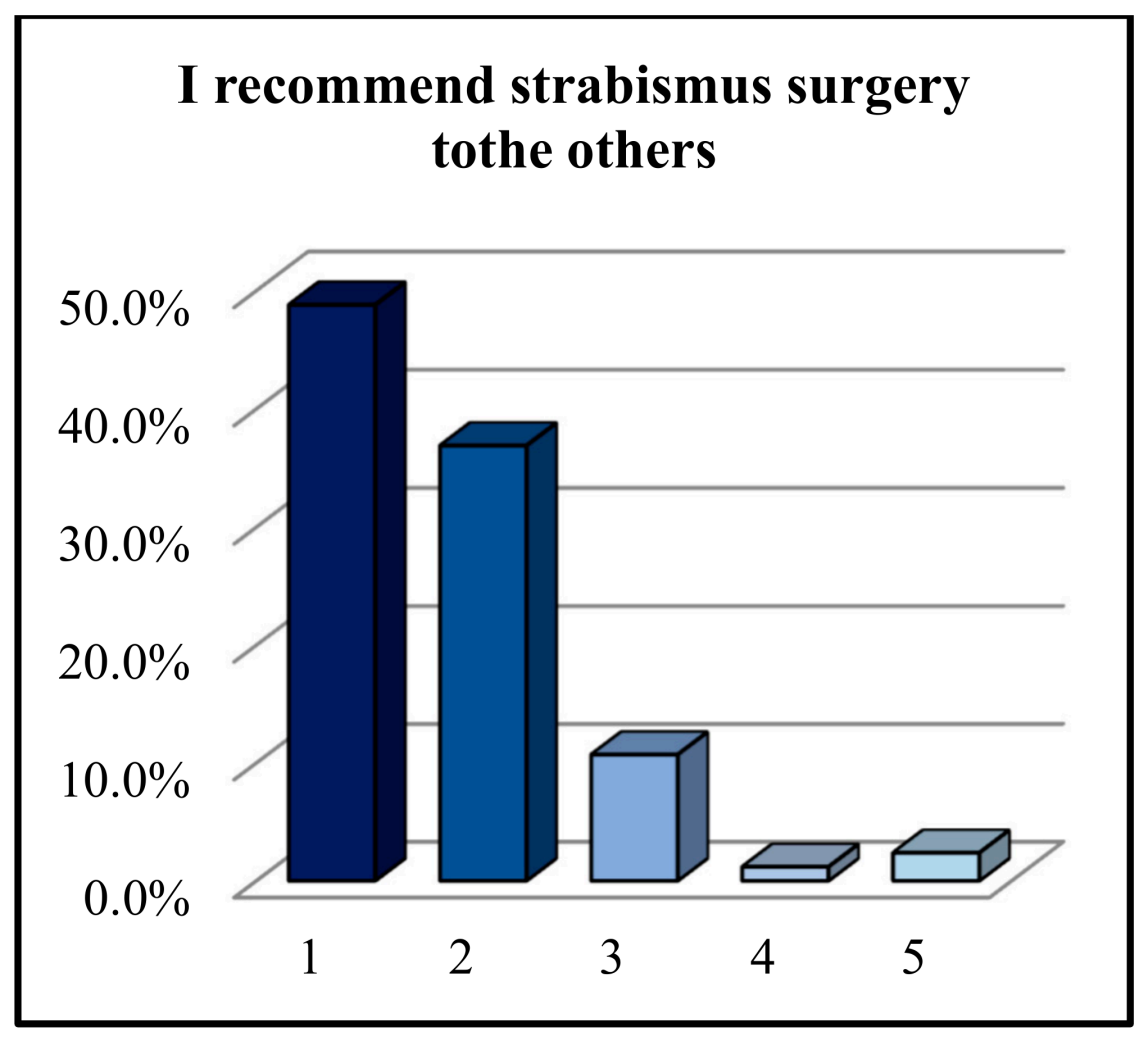
Recommending strabismus surgery to others. 1: Totally agree; 2: Agree; 3: Not sure; 4: Disagree; 5: Totally disagree.

A total of 116 children met the eligibility criteria and were enrolled in the study. Of these, 84 participants (42 boys and 42 girls) had complete preoperative and postoperative PHSCS assessments together with complete ophthalmological records and were included in the final statistical analysis (Table 1). Thirty-two participants were excluded because of incomplete clinical records and/or missing postoperative questionnaire data, precluding paired analysis. The mean age of the analyzed cohort was 10.9 ± 2.2 years. The percentage of kids who said that strabismus affects their lives was 73.6%, and the majority of them said it has a negative impact (77.4%) (Figure 1). A percentage of 65.5% reported that their condition affects their friendships, and 65.4% said it has negative effects (Figure 2). A percentage of 80.9% were pleased with the outcome of their surgery, and 85.7% recommended it to others (Figure 3 and Figure 4). The self-concept scale average scores before and after surgery were 58.2 ± 10.4 and 59.0 ± 10.1, with a *p*-value of 0.05 (Table 2). The ‘Physical Appearance and Attributes’ subscale showed a statistically significant positive change postoperatively (8.3 ± 1.6 and 8.9 ± 1.9, respectively, *p* < 0.005), while other subscales did not change.

## 4. Discussion

Disruption of ocular alignment, which plays a crucial role in nonverbal human communication, can significantly impair interpersonal interactions. The eyes are among the most expressive and socially significant features of the face; thus, any misalignment can disrupt effective communication, hinder emotional expression, and impede the ability to establish eye contact. Strabismus has psychological effects that extend beyond its classification as merely a cosmetic or medical issue requiring correction.

Studies indicate that many adults perceive strabismus as a serious issue that affects their interactions with others, their job prospects, and their social lives. However, there is a lack of surveys that allow children to share their perspectives on this matter.

In a study comparing the psychosocial effects of strabismus on adults and children, Bandhu et al. found that strabismus had a negative psychosocial impact on both groups [11]. Still, the nature and extent of the psychosocial impact differed according to age. Strabismus causes problems with interpersonal relationships to a lesser extent in children (37.5%) than in adults (75%). Children (37.5%) also reported feeling less disadvantaged in terms of available opportunities compared to adults (95.83%) [12,13]. This study focused on children aged 5 to 18; however, our research specifically examined younger kids, from late childhood to early adolescence (ages 7 to 15). We excluded high school students because their psychosocial dynamics differ significantly from those of elementary school students.

We aimed to study the younger ages where the self-concept, self-esteem, and social skills are still developing. Previous studies claim that self-esteem is not fixed in middle childhood: it increases until around age 11 and remains in flux through early adolescence (≈11–15 years) as cognitive, social, and emotional processes continue to evolve. Therefore, it is crucial to distinguish between distinct psychological stages in children.

Another study has shown that adults with strabismus are more concerned about how they appear to others, eye contact, and interpersonal relationships [14].

The increasing responsibilities and expectations might also affect the psychology of adults. Studies report that adults with strabismus may face reduced employment opportunities and lower employability prospects compared to their peers [15]. Compared to children (56.25%), adult patients think more frequently about their strabismus, which affects their concentration (100%) [11]. Other studies have reported that the psychosocial impact of strabismus is more intense in adults than in children. One study reported that the difficulties faced by individuals with strabismus do not disappear after childhood but rather intensify during adolescence and adulthood. These individuals experienced higher levels of distress overall than age- and sex-matched controls (*p* < 0.01) [16].

In our study, only 42.7% of the children thought that their strabismus negatively affected their communication with their friends. However, 80.9% reported that they were happy after the surgery, and 85.7% stated that they would recommend strabismus surgery to other people.

Taken together, our findings suggest that strabismus surgery may have a positive influence on children’s perception of their physical appearance and overall satisfaction. However, given the absence of a control group, these findings should not be interpreted as evidence that surgery prevents adverse psychosocial outcomes. Rather, our results indicate that surgical correction may contribute to improvements in selected domains of self-concept in children who require surgical treatment.

Silva et al. evaluated functional vision and Eye Related QoL (ERQOL) in children with strabismus and their parents with the PedEyeQ questionnaire and reported that scores were significantly lower than the control group [17]. Furthermore, even those who underwent successful strabismus surgery or surgeries had significantly lower scores compared to visually healthy children. However, 38% of the participants in the strabismus group were under 4, and they did not fill out the children’s part of the questionnaires. So, it might affect the results since the parents’ opinions and worries about their kids might not reflect the kids’ self-concept. That is why, in this study, we included literate school kids who can fill in the questionnaires by themselves.

Temeltürk et al., in their study evaluating the psychosocial effects of strabismus surgery on children with strabismus and their parents, reported that parents’ depression scores were higher before surgery compared to the control group, and that the scores decreased after surgery [6]. They reported that the surgery reorganized the social and emotional worlds of the child and parents.

It is not clear if the self-concept improvement in the children after surgery has any correlation with parents’ depression scores. In our study, even if the self-reported self-concept scores were above average in strabismic kids, there was a slight improvement in the appearance subscale after surgery.

This study has several limitations. First, the absence of a non-surgical control group precludes conclusions regarding causality or the long-term protective effect of surgery on self-concept. Second, detailed clinical characteristics, including the duration of strabismus, binocular vision status, amblyopia, refractive error, and other disease-specific variables, were not analyzed in relation to self-concept outcomes. Third, objective postoperative ocular alignment and surgical success were not evaluated as outcome measures; therefore, correlations between motor outcomes and psychosocial improvement could not be assessed. Finally, multiple comparisons were performed across the PHSCS subscales without formal adjustment for multiplicity. Consequently, the subscale analyses should be considered exploratory, and the statistically significant improvement observed in the Physical Appearance and Attributes domain should be interpreted with appropriate caution. Future prospective studies incorporating detailed clinical parameters, objective surgical outcomes, and control groups are warranted to validate these findings.

Despite these limitations, this study provides insight into children’s own perceptions of strabismus by using a validated self-concept instrument before and after surgery. These findings highlight that the benefits of strabismus surgery may extend beyond ocular alignment and include aspects of children’s perceived physical appearance. Incorporating patient-reported outcome measures into future studies may improve the understanding of psychosocial well-being following strabismus treatment.

## 5. Conclusions

School-aged children with strabismus may experience psychosocial difficulties related to their condition. In this prospective study, strabismus surgery was associated with improved self-perception of physical appearance, whereas the overall self-concept remained largely stable. These findings suggest that surgical correction may contribute to positive psychosocial outcomes in appropriately selected patients; however, larger prospective controlled studies are required to confirm these observations.

## Figures and Tables

**Table 1:** Demographics and survey questions, and answers.

		Min–Max	Median	Mean/n-%			I.Q–3.Q
Age		7.0–15.0	11.0	10.9 ± 2.2			9.0–12
Gender	Male			42		50.0%	
	Female			42		50.0%	
Does being strabismic affect your life?	No			22		26.2%	
	Yes			62		73.8%	
Affect negatively							
Totally agree				27		43.5%	
Agree				21		33.9%	
Not sure				5		8.1%	
Disagree				8		12.9%	
Totally disagree				1		1.6%	
Does being strabismic affect your relationship with friends?	No			29		34.5%	
	Yes			55		65.5%	
Affect negatively							
Totally agree				18		32.7%	
Agree				18		32.7%	
Not sure				5		9.1%	
Disagree				9		16.4%	
Totally disagree				5		9.1%	
I am satisfied with the result of my surgery	Totally agree			37		44.0%	
	Agree			31		36.9%	
	Not sure			15		17.9%	
	Totally disagree			1		1.2%	
Recommending strabismus surgery to others	Totally agree			41		48.8%	
	Agree			31		36.9%	
	Not sure			9		10.7%	
	Disagree			1		1.2%	
	Totally disagree			2		2.4%	

**Table 2:** Evaluation of self-concept scale before and after strabismus surgery.

	Preoperative		Postoperative		*p*
	Mean	Median	Mean	Median	
Self-concept Scale					
Behaviour	11.5 ± 2.3	12.0	11.6 ± 2.4	12.0	0.249 ^W^
School Status	14.1 ± 2.4	15.0	14.2 ± 2.2	15.0	0.485 ^W^
Physical App + Attributes	8.3 ± 1.6	8.0	8.9 ± 1.9	9.0	0.005 ^W^
Anxiety	14.6 ± 2.9	15.0	14.9 ± 2.8	15.5	0.138 ^W^
Popularity	9.3 ± 2.3	10.0	9.4 ± 2.2	9.0	0.335 ^W^
Happiness + Satisfaction	8.6 ± 1.9	9.0	8.7 ± 1.9	9.0	0.126 ^W^
Total Score	58.2 ± 10.4	60.0	59.0 ± 10.1	60.0	0.076 ^W^

^w^ Wilcoxon test.

## Data Availability

The raw data supporting the conclusions of this article will be made available by the authors on request due to ethical restrictions.
